# Combinatory local ablation and immunotherapies for hepatocellular carcinoma: Rationale, efficacy, and perspective

**DOI:** 10.3389/fimmu.2022.1033000

**Published:** 2022-11-23

**Authors:** Shuling Chen, Xuezhen Zeng, Tianhong Su, Han Xiao, Manxia Lin, Zhenwei Peng, Sui Peng, Ming Kuang

**Affiliations:** ^1^ Institute of Diagnostic and Interventional Ultrasound, The First Affiliated Hospital, Sun Yat-sen University, Guangzhou, Guangdong, China; ^2^ Center of Hepato-Pancreato-Biliary Surgery, The First Affiliated Hospital, Sun Yat-sen University, Guangzhou, Guangdong, China; ^3^ Institute of Precision Medicine, The First Affiliated Hospital, Sun Yat-sen University, Guangzhou, Guangdong, China; ^4^ Department of Oncology, The First Affiliated Hospital of Sun Yat-sen University, Guangzhou, Guangdong, China; ^5^ Department of Radiation Oncology, The First Affiliated Hospital of Sun Yat-sen University, Guangzhou, Guangdong, China; ^6^ Zhongshan School of Medicine, Sun Yat-sen University, Guangzhou, Guangdong, China

**Keywords:** ablation, tumor immune microenvironment, immunotherapy, HCC, combination therapy

## Abstract

Hepatocellular carcinoma (HCC) is the leading cause of cancer-related death worldwide. Local ablation, such as radiofrequency ablation, microwave ablation, cryoablation and irreversible electroporation, etc., are well established in elimination and control of HCC. However, high recurrence rate after local ablation remains the biggest challenge for HCC management. Novel and effective therapeutic strategies to improve long-term survival are urgently needed. Accumulating studies have reported the role of ablation in modulating the tumor signaling pathway and the immune microenvironment to both eliminate residual/metastatic tumor and promote tumor progression. Ablation has been shown to elicit tumor-specific immune responses by inducing massive cell death and releasing tumor antigen. Immunotherapies that unleash the immune system have the potential to enhance the anti-tumor immunity induced by ablation. Multiple combinatory strategies have been explored in preclinical and clinical studies. In this review, we comprehensively summarize the latest progress on different mechanisms underlying the effects of ablation on tumor cells and tumor microenvironment. We further analyze the clinical trials testing the combination of ablation and immunotherapies, and discuss the possible role of immunomodulation to boost the anti-tumor effects of ablation and prevent HCC recurrence.

## Introduction

Liver cancers rank as the sixth most common cancers and the fourth leading cause of cancer-related death, and remain to be one of the few human malignancies still trending upwards worldwide (1, 2). Nevertheless, with the advance of screening technology and increased awareness of cancer surveillance, more and more HCC could be detected at early stage, rendering curative therapeutics applicable. In the 2022 update of BCLC strategy for HCC management, local ablation still plays leading part among the recommended curative treatments for early-stage HCC (3). A vast range of percutaneous ablation techniques have changed over the past decades, enabling improved local control efficacy for more and more HCC patients. Study showed that approximately 10% of HCC tumors ≤ 2 cm developed intrahepatic metastasis, and about 27% of these tumors developed microvascular invasion, which leads to repeated recurrence in many HCC patients (4). Thus, adjuvant therapies that could prevent HCC recurrence after curative treatment could dramatically improve the prognosis. Of note, in the era of immunotherapy, ablative techniques are gaining more and more attention for their capability of boosting local and systemic immune effects, which makes combination strategy a promising weapon for HCC treatment. Herein, we summarized the current status and progress of various ablation and immunotherapy for HCC, discussed the rationale for their synergistic anti-tumor effects, and conceived the current trends and future prospects of their combination, hoping to shed light on future studies for ablative immunotherapy to yield a promising new era of HCC management.

Copious ablation therapeutics are feasible in clinic practice, among which radiofrequency ablation (RFA) remains the backbone of local ablation for early-stage HCC. Other ablative techniques including microwave ablation (MWA), cryoablation and irreversible electroporation (IRE) are also available for various HCC cases. However, more data is needed for other ablation choices to become the mainstay treatments of HCC. By and large, two indications for these ablative therapies are referred by guidelines, as first pick for single, very early tumors < 2 cm or as a substitute to resection in early-stage single tumors ≤ 4 cm, or 2–3 tumors ≤ 3 cm (5, 6). Typically, ablation destroys tumor by chemical, electrical or thermal technologies. RFA, MWA, laser and high intensity focused ultrasound (HIFU) ablation deliver focal hyper thermic injury to tumor cells (7, 8). Cryoablation (CRA) causes hypo thermic damage to ablated cells while IRE is a non-thermal ablative technique that destroys cell by changing cell permeability (8). Chemical ablations mostly use ethanol and acetic acid injections (9).

## Effects of ablation on HCC

This section presents a brief retrospect of traditional ablative therapies for HCC, as well as newly emerging ablative techniques, and discusses their traditional anti-cancer effects.

### Radiofrequency ablation

RFA is the most widely adopted local ablation therapies for HCC because of its superiority to other ablative treatment in objective response rates and overall survival (10, 11). Moreover, survival rate of RFA is comparable to that of surgical resection in stratified patients (12). Being repeatable, more cost-effective, and less invasive, RFA has been recommended as the first-line therapy for early-stage HCC by AASLD and EASL guidelines (5, 6). Complete response rates range from 70% to 90% and a median overall survival of ~60 months have been reported (13, 14). Percutaneous RFA is performed by direct insert of electrodes into the tumor tissue under the guidance of ultrasound, computed tomography (CT) or magnetic resonance. High-frequency alternating current at 375–480 kHz from the electrodes generates temperatures between 60°C and 100°C to yield tumor necrosis (15). Traditional monopolar RFA is limited in tumors ≤ 2–3 cm or near vessels (16). Cytotoxic temperatures are hard to maintain when the ablated tumor is near large blood vessels because flowing blood would adsorb the heat energy, which is called heat sink effect^6^. Innovative techniques including multibipolar RFA are developed to improve ablation efficacy.

RFA destructs HCC cells by inducing hyperthermic injury, which causes rapid protein denaturation, cell membrane integrity loss, mitochondrial dysfunction, and inhibition of DNA replication (17, 18). In addition, indirect or delayed cellular damage play important parts in tumor damage after thermal ablation. Potential mechanisms includes induction of apoptosis, ischemia after vascular damage, ischemia–reperfusion injury, and release of lysosomal contents and cytokines from tumor cells and intruding inflammatory cells to stimulate further immune response (17).

However, high recurrence of HCC after RFA has been reported, with a 5-year recurrence rate of 50–70%, for which insufficient RFA (iRFA) is mainly to blame^1^. During RFA, three zones could be detected in heat-ablated lesions: central zone suffers from coagulative necrosis with temperature ≥ 50°C; transitional zone is exposed to sublethal heat stress and induces reversible cell damage; the surrounding liver tissue that is unaffected by sublethal heat^6^. iRFA endows HCC with a more malignant phenotype, leading to drug resistance and worse prognosis. Several mechanisms have been reported involved in HCC recurrence after iRFA. Activation of b-catenin, Akt, ERK1/2, HIF-1a/BNIP3, MAPK, and NF-kB signaling pathways as well as inhibition of STAT3 signaling pathways have been demonstrated to promote HCC progression after iRFA (19–25). Besides, ceRNA-mediated mechanisms including ASMTL-AS1/miR-342-3p/NLK/YAP axis and GAS6-AS2/miR-3619-5p/ARL2 axis are also uncovered (26, 27). Kuang’s team recently reported transcription and translation regulatory mechanisms working in HCC cells after iRFA. The sublethal heat treatment increased the level of stress-induced phosphoprotein 1 (STIP1) and heat shock protein 90 (HSP90), and promoted the formation of STIP1-HSP90 complex, which transferred epithelial transcription suppressor Snail1 into nucleus to modulate mesenchymal gene transcription (28). In addition, sublethal heat stress increased the m6A epigenetic modification of epidermal factor growth receptor (EGFR) and promoted its binding with YTHDF1, which enhanced the translation of EGFR mRNA, leading to the migration and invasion of HCC cells (29).

Endeavors to combat iRFA have been devoted recent years. Nanotechnology and artificial intelligence (AI) based radiomics have advanced greatly to counter iRFA. Deep learning radiomics improve the accuracy of imaging guided identification of ablation tumor boundaries and the accurate preoperative prediction of prognosis for RFA and surgery, facilitating the optimized decision making between them for HCC patients in early stage. Liu et al. retrospectively enrolled 419 patients examined by contrast-enhanced ultrasound (CEUS) within 1 week before RFA or surgical resection. The nomograms incorporating radiomics signatures and clinical variables for progression-free survival (PFS) prediction were built. The proposed radiomics models and nomograms yielded accurate preoperative prediction of PFS for RFA and liver resection (30). Jiang et al. designed a nanobubble conjugated with colony-stimulating factor 1 receptor (CSF-1R), called NBCSF-1R, for HCC margin detection, facilitating the determination of ablation margin (31). Further, the combination of systemic or immunotherapy would be a promising sally port to overwhelm HCC recurrence after iRFA.

### Microwave ablation

MWA generates heat through electromagnetic waves with higher frequency (900–2,450 MHz), endowing it with several advantages over RFA, including higher temperature for larger ablation zone, shorter ablation time, and a lower susceptibility to heat-sink effects (32). MWA created electromagnetic field to force the polar molecules with intrinsic dipoles including predominantly water within the tissue to keep realigning with the oscillating electric field (32). MWA also destroy tumor cells *via* the aforementioned heat-ablated mechanisms of direct hyperthermic damage. However, MWA-induced pro-inflammatory cytokines including IL-1 and IL-6 is less compared with that from the other ablative technologies (33). Several trials found comparable treatment efficacy of MWA to RFA by reporting similar primary endpoint and local tumor progression at 2 years (33). However, phase III data is needed for recommending this treatment in early-stage HCC with high level of evidence.

### Irreversible electroporation

IRE works through non-thermal manner. It delivers short electric pulses of high-voltage field current between two inserted electrodes and punches the cellular bilipid membrane to induce cell apoptosis (34). Its non-thermal mechanism lowers the risk of heat injury to the adjacent tissue. For this, heat sink effect poses little influence on the efficacy of IRE ablation. Therefore, IRE is better suitable for HCC located at risk anatomical position. Jean-Charles Nault et al. treated 58 patients with IRE and reported a complete ablation rate of 92%, and 70% of the cases got tumor progression-free survival at one year (35). In general, IRE could be an alternative for HCC not suitable for thermal ablation. Similar as WMA, large cohorts of patients with longer follow-up are needed to evaluate the long-range treatment efficacy of IRE.

### Other ablative techniques

Real world data is limited for other ablative techniques to date, including cryoablation, laser ablation and phototherapy. A multi-center RCT comparing RFA and CRA observed comparable results in overall survival and tumor-free survival while a retrospective study with large cohort reported greater advantage of CRA in HCC-specific survival in comparison to RFA (36, 37). Phototherapy including photodynamic therapy (PDT) and photothermal therapy (PTT) is a novel and promising cancer therapy. Phototherapy destroys tumor cells through photochemical or photophysical effects (38). Nevertheless, these techniques are way far from recommendation in daily clinical practice.

## Effects of ablation on tumor immune microenvironment

Accumulating studies have demonstrated the effect of ablation in shaping the immune microenvironment. Tumor neoantigen, cytokines and danger-associated molecular patterns (DAMPs) induced by ablative therapies are recognized as the source of immune activation (7, 39). Besides, ablation will also trigger physiological wound healing response that regulates and maintains immunological tolerance towards the damaged tissue (39). Different ablative therapies triggered various immune responses in the tumor immune microenvironment.

### Immune responses induced by RFA

Among all the ablation therapies, RFA has been the most widely used percutaneous ablation in early-stage HCC. To investigate the dynamic changes of systemic immunity in HCC patients after RFA treatment, Rochigneux et al. collected PMBCs of 80 patients on the day before (D0), day after (D1) and month after RFA, and detected the frequencies and phenotypes of different immune cells. They found that an early dynamic (D0/D1) of activated NKp30^+^ natural killer (NK) cells was associated with decreased recurrence, while a late dynamic (D1/M1) of immature CD56^bright^ NK cells and altered PD-L1^+^myeloid-derived dendritic cells (DCs) correlated with increased recurrence (40). Another study also showed that RFA treatment stimulated NK cell activation and differentiation, and the number of NK cells with high level of activatory NK receptors and enhanced cytotoxicity were significantly increased in peripheral blood of HCC patients after RFA treatment (41). In addition, CD4^+^ and CD8^+^ T cell response induced by ablation correlated with clinical outcomes (42, 43). Although RFA could enhance various tumor-associated antigens (TAA) -specific T cell responses which contributed to improved recurrence-free survival, this effect was not sufficient to prevent HCC recurrence completely due to the short lifetime of TAA-specific T cells (44, 45). In addition, the number of TAA-specific T cells was inversely correlated with the frequency of CD14^+^HLA-DR^-/low^ monocytic myeloid-derived suppressor cells (M-MDSCs), suggesting the immunosuppression in tumor immune microenvironment (44). Similarly, another study showed that in post-RFA recurrent HCC, polymorphonuclear myeloid-derived suppressor cells (PMN-MDSCs) were accumulated in the tumor microenvironment to suppressed CD8^+^T cells, providing the immunosuppressive soil for tumor progression (46). Mechanistically, RFA-mediated heat treatment-induced methyltransferase 1 (METTL1) overexpression, which subsequently translationally upregulated transforming growth factor-beta 2 (TGF-β2) to induced PMN-MDSCs and suppressed CD8^+^T cell proliferation (46).

In addition to cytotoxic lymphocytes, accumulating studies have shown that ablation treatment could induce DC infiltration in tumor and activate DCs to evoke anti-tumor immune responses (47–49). Increased serum levels of tumor necrosis factor-alpha (TNF-α), interleukin-1β (IL-1β), interferon-gamma (IFN-γ), and IL-2 were also observed, while the levels of Th2 cytokines including IL-4, IL-6 and IL-10 were markedly decreased (50, 51).

Vascular endothelial growth factor (VEGF) is an angiogenic factor that regulates angiogenesis by inducing proliferation, migration and permeability of endothelial cells. It has been reported that VEGF was increased in HCC patients after RFA treatment (52). VEGF also play a immunoregulatory role in tumor microenvironment by inducing MDSCs, regulatory T cells (Tregs), and mast cells, and inhibiting T cell function, differentiation and activation of DCs (53, 54).

### Immune responses induced by other ablation therapies

As for other ablation therapies, cryoablation could induce inflammatory and coagulative responses in liver (55). Interestingly, study also found that elevation of circulating PD-L1/PD-1 in hepatitis B (HBV)-associated HCC patients after cryoablation correlated with poor prognosis (56). MWA significantly increased CD3^+^T cells, CD4^+^T cells and IL-2 in peripheral blood of HCC patients one month after treatment. In addition, IL-4 and IL-10 levels were decreased after MWA, indicating that MWA relieved immunosuppression in HCC patients (57). Besides, MWA increased T helper 17 cells (Th17) in HCC patients, and high frequency of circulating Th17 cells was associated with tumor recurrence (58). Study also found that the tumor-specific T cell response against TAAs was more frequent in patients with a long-time remission (> 1 year) after MWA compared to patients suffering from an early relapse, and correlated with improved PFS (59). Irreversible electroporation not only effectively eliminated HCC but also prevented tumor recurrence (60). On the one hand, IRE induced tumor cell necrosis and release of DAMPs including adenosine triphosphate (ATP), high mobility group box 1 (HMGB1) and calreticulin to stimulate anti-tumor immunity. On the other hand, IRE also alleviated immunosuppression by reducing Tregs and PD-1^+^ T cells (60).

Substantial evidence phenotypically showed that ablation induced immune cell changes and differential inflammatory cytokines and chemokines expression in peripheral blood or tumor microenvironment. In addition, ablation induces both protective anti-tumor immune response and immune tolerance. However, most studies have not yet explored the underlying mechanisms. It’s conceivable that DAMPs or inflammatory cytokines or chemokines induced by ablation may play the pivotal role in reshaping the immune microenvironment. Indeed, a few mechanistic studies reported that ablation affects the immune microenvironment indirectly through the regulation of cytokines or chemokines expression by residual tumor cells (46, 60). However, the complex regulatory network by ablation awaits further investigation in the future. Currently, efforts have been focused on developing adjuvant immunotherapy to synergistically shift the equilibrium out of inhibitory immune modulation and elicit sustainable immune response against tumor.

## Combination of ablation and immunotherapy

All ablation modalities generate tumor debris *in situ*, which provides antigen depot also known as cancer vaccine to stimulate immature DCs and naïve T cells that evoke antitumor immunity (7, 39). However, immune responses induced by ablative treatment are incapable of evoking robust sustainable immune effects. In addition, tumors evolved to create an immunosuppressive tumor immune microenvironment favorable for tumor progression. Developing approaches that counteract the immunosuppressive microenvironment potentially boost ablation-induced anti-tumor immune response (39).

Immunotherapy, either unleashes the own immune system or adoptively transfers cytotoxic cells to fight cancers provides the rationale for combination therapy. Different immune strategies have been tested in many studies, including adoptive cell therapy, immune checkpoint inhibitors (ICI), cytokines, *etc.* Currently, immunotherapies have been mostly used in advanced diseases, it’s also reasonable to use them in curative and adjuvant setting. Many clinical trials have been launched to investigate the safety and efficacy of combination of ablation and immunotherapy in HCC (**Table 1**).

**Table 1:** Clinical trials evaluating the combination of ablation and immunotherapy in HCC.

Trial ID	Phase	Study population	Drug	Recruitment status
NCT03847428	III	patients with HCC who are at high risk of recurrence after curative hepatic resection or ablation	durvalumab in combination with bevacizumab or durvalumab alone	Active, not recruiting
NCT04150744	II	advanced HCC	RFA +PD-1 immunosuppressant (carrizumab) or carrizumab alone	Recruiting
NCT03337841	II	HCC before and after curative surgery or ablation	Pembrolizumab	Unknown
NCT03753659	II	HCC patients who are candidates for local ablation *via* either RFA or MWA or brachytherapy	Pembrolizumab or TACE	Recruiting
NCT03630640	II	advanced HCC treated by curative electroporation	Nivolumab	Active, not recruiting
UMIN000026648	II	HCC patients who showed a complete response after resection or RFA	Nivolumab	Completed
NCT03383458	III	HCC patients who have undergone complete resection or have achieved a complete response after local ablation, and who are at high risk of recurrence	Nivolumab	Active, not recruiting
NCT03867084	III	HCC patients who have undergone complete resection or complete local ablation	Pembrolizumab	Recruiting
NCT04102098	III	HCC patients who have undergone complete resection or have achieved a complete response after local ablation, and who are at high risk of recurrence	atezolizumab plus bevacizumab	Active, not recruiting
NCT02821754	II	HCC or Biliary Tract Carcinomas (BTC)	Durvalumab + Tremelimumab	Active, not recruiting
NCT04220944	I	Unresectable HCC treated with MWA combined with simultaneous TACE	Sintilimab	Recruiting
NCT03939975	II	advanced HCC	pembrolizumab or nivolumab or JS001	Completed
NCT03864211	I/II	unresectable HCC	Toripalimab	Active, not recruiting
NCT04652440	II	HCC	Tirelizumab	Recruiting
NCT01853618	I/II	advanced HCC	Tremelimumab	Completed

### Adoptive cell therapy

Adoptive cell therapy is an immunotherapy that uses autologous immune cells which are modified or activated to evoke anti-tumor immunity and eliminate tumor cells. Cytokine-induced killer cells (CIK), tumor-infiltrating lymphocytes (TIL), chimeric antigen receptors (CAR)-T cells, DC vaccines are common forms of adoptive cell therapy.

CIK cells are a heterogeneous population of effector T cells, which come from patients’ peripheral blood mononuclear cells and can be expanded *in vitro*. CIK alone has been developed as cancer immunotherapy as it exhibits major histocompatibility complex (MHC)-unrestricted, safe, and effective anti-tumor activity (61). In the recent years, CIK has also been investigated as adjuvant therapy in treatment of HCC. Adjuvant therapy using cytokine-induced killer cells are derived from peripheral blood mononuclear cells (PBMCs) of HCC patients and activated by IL-2 and anti-CD3 antibody. To test the safety and feasibility of combination of RFA and adjuvant autologous RetroNectin activated killer (RAK) cells, 7 HCC patients were recruited in the trial. RAK cells were transfused to primary HCC patients intravenously after RFA. During a seven-month follow-up, no severe adverse events, recurrences or deaths were observed, suggesting the feasibility and safety of the combined therapeutic strategy to prevent HCC recurrence (62). In 2008, Weng et al. launched a clinical trial which recruited 85 HCC patients after transcatheter arterial chemoembolization and RFA. Autologous CIK cells were transfused to patients *via* hepatic artery. After infusion, CD3^+^, CD4^+^, CD56^+^, CD3^+^CD56^+^ cells, and CD4^+^/CD8^+^ ratio were significantly increased, resulting in a reduction of HCC recurrence (63). A study in Korea recruited 230 patients with HCC treated by surgical resection, radiofrequency ablation, or percutaneous ethanol injection, and patients were administered with control or CIK therapy respectively. The median time of recurrence-free survival was significantly improved from 30.0 months to 44 months (64). In another clinical trial, 62 patients diagnosed with HCC were treated with RFA alone or combined with CIK. The combination of sequential CIK with RFA improved progression-free survival, and reduced Hepatitis C (HCV) viral load in some patients (65). Consistently, a similar clinical trial also showed that autologous CIK cells after RFA treatment prolonged the RFS of HCC patients (66). These positive clinical results suggested the potential of this combined treatment in prevention of HCC recurrence (65).

The DC and T cell adoptive transfer have also been studied in HCC. In a phase II clinical trial, Peng et al. investigated the combination of neoantigen-based DC vaccination and adoptive T-cell transfer as adjuvant therapy after RFA or surgical resection of HCC patients. This combination therapy successfully induced neoantigen-specific immunity and prolonged disease-free survival in responders without severe side effects, indicating that neoantigen-based combination immunotherapy is feasible, safe, and has the potential to reduce HCC recurrence after curative treatment (67).

### Immune checkpoint inhibitor

The development of ICIs has revolutionized the treatment of cancers and provides unprecedented extension of patient survival. Immune checkpoints are common part of the immune system which regulate T cell activity. ICIs achieved great success in treating various types of solid and liquid malignancies. ICIs work by releasing the inhibitory brakes of T cells and also activating other innate and adaptive immune cells, which orchestrate an effective immunity to eliminate tumors (68). Many studies have reported the positive results of ICIs as the first-line treatment in advanced cancers. And increasing studies have been investigating the efficacy of ICIs as combination with curative treatment (listed in **Table 1**).

In some trials, positive clinical results have been observed in advanced HCC patients who received cytotoxic T-lymphocyte-associated protein 4 (CTLA4) blockade (Tremelimumab) combined with RFA, accompanied by remarkably reduced HCV viral load. In addition, the combined treatment increased CD8^+^T cells infiltration in tumor (69). However, some of these patients also received cryoablation and/or transarterial chemoembolization (TACE). In another study, it showed that RFA induced anti-tumor immune response which was strongly enhanced by CTLA4 blockade, contributing to long-lasting tumor protection (70). For advanced HCC patients, anti-PD-1 (programmed cell death protein 1) antibody is one of the second-line therapies after sorafenib failure. Lyu et al. found that additional ablation increased response rate with tolerable toxicity and improved survival in these patients (71). Many trials are still under investigation and results of these trials will be public within the next few years.

In preclinical model, Huang et al. reported that the combination of MWA and anti-PD-1 antibody significantly ameliorated distant tumor growth and elevated Th1 cytokines in peripheral blood in mouse HCC model (72).

### Other immunotherapies

Multiple studies also investigated the efficacy of other adjuvant immunotherapies after ablation. CpG B oligonucleotides, a toll like receptor 9 agonists, has been tested in a rabbit VX2 hepatoma model. It showed that RFA alone could induce the secretion of Th1 cytokines, while CpG treatment increased IL-8 and IL-10 levels. In addition, the combination of CpG and RFA significantly reduced tumor burden and even prevented subsequent tumor metastasis, and thus improved survival (73).

Several chemokines were also used in cancer treatment for their ability to attract immune cells such as DC and cytotoxic T cells to augment anti-tumor immunity. It was reported that RFA alone could not only eradicate the treated ipsilateral tumors, but also inhibited the growth of contralateral non-RFA-treated tumors with increased T-cell infiltration. Additional injection of ECI301 (an active variant of CC chemokine ligand 3) after RFA significantly augmented RFA-induced anti-tumor immune responses and increased CCR1-expressing CD11c^+^ cells in peripheral blood and tumors. Deficiency of CCR1 impaired the accumulation of CD11C^+^, CD4^+^ and CD8^+^ cells in tumors, indicating that ECI301 augmented tumor-specific immune responses through CCR1-mediated DC accumulation in tumor (74).

## Perspective/conclusive remarks

The therapeutic strategies of HCC are evolving rapidly. The rationale for combination of ablation and immunotherapy is sound. Ablation promotes production of proinflammatory cytokines and antigens, which activated anti-tumor immunity. Tyrosine kinase inhibitors (TKIs) or immunotherapies, which modulates the tumor microenvironment by increasing effector T cell infiltration and decreasing immunosuppressive cells, further enhances anti-tumor immune response.

However, some issues need to be further investigated, including the timing of ICB administration, biomarkers that could predict therapeutic effects and management of adverse events. Immune profiling after ablation is essential for development of combination therapies that boost anti-tumor immunity (75).

In the recent years, various types of immunotherapies have come to the stage of HCC treatment, such as Lymphocyte-activation gene 3 (LAG-3), T cell Ig and ITIM domain (TIGIT) or T cell immunoglobulin domain and mucin domain-3 (TIM-3) blockade, chimeric antigen receptor T cell (CAR-T) therapy, adoptive cell therapies using NK cells, NKT cells, or γδ T cells, oncolytic virotherapy, cancer vaccines, *etc (* 76, 77). These novel therapeutic strategies also show great potential to synergize with ablation in the treatment of primary and metastatic HCC. Increasing clinical trials are actively underway and may offer a paradigm shift in the management of HCC.

## Author contributions

SC, XZ and TS wrote the manuscript. SC, HX, ML, ZP, SP, MK revised this manuscript. All authors contributed to the article and approved the submitted version.

## Funding

This work is supported by State Key Program of National Natural Science of China (No. 82130083), The National Science Fund for Distinguished Young Scholars (No. 81825013), the National Science Fund for Young Scholars (No. 82003105), the National Natural Science Foundation of China (No. 82172047; No. 31970870), Natural Science Foundation of Guangdong Province (No. 2021A1515010450), Natural Science Foundation for Distinguished Youths of Guangdong Province (No. 2022B1515020060), the Joint Funds of the National Natural Science Foundation of China (No. U20A20370).

## Conflict of interest

The authors declare that the research was conducted in the absence of any commercial or financial relationships that could be construed as a potential conflict of interest.

## Publisher’s note

All claims expressed in this article are solely those of the authors and do not necessarily represent those of their affiliated organizations, or those of the publisher, the editors and the reviewers. Any product that may be evaluated in this article, or claim that may be made by its manufacturer, is not guaranteed or endorsed by the publisher.
